# Association of Acute Postoperative Pain and Cigarette Smoking With Cerebrospinal Fluid Levels of Beta-Endorphin and Substance P

**DOI:** 10.3389/fnmol.2021.755799

**Published:** 2022-01-10

**Authors:** Fan Wang, Hui Li, Qingshuang Mu, Ligang Shan, Yimin Kang, Shizhuo Yang, Hui-Chih Chang, Kuan-Pin Su, Yanlong Liu

**Affiliations:** ^1^Beijing Hui-Long-Guan Hospital, Peking University, Beijing, China; ^2^Key Laboratory of Psychosomatic Medicine, Inner Mongolia Medical University, Huhhot, China; ^3^Department of Biomedical Engineering, College of Engineering, Peking University, Beijing, China; ^4^Xinjiang Key Laboratory of Neurological Disorder Research, The Second Affiliated Hospital of Xinjiang Medical University, Urumqi, China; ^5^Department of Anesthesiology, The Second Affiliated Hospital of Xiamen Medical College, Xiamen, China; ^6^Department of Anesthesiology, The Affiliated Hospital of Inner Mongolia Medical University, Huhhot, China; ^7^School of Pharmaceutical Sciences, Wenzhou Medical University, Wenzhou, China; ^8^Department of Psychiatry & Mind-Body Interface Laboratory (MBI-Lab), China Medical University Hospital, Taichung, Taiwan; ^9^College of Medicine, China Medical University, Taichung, Taiwan; ^10^An-Nan Hospital, China Medical University, Tainan, Taiwan; ^11^The Affiliated Kangning Hospital, Wenzhou Medical University, Wenzhou, China; ^12^School of Mental Health, Wenzhou Medical University, Wenzhou, China

**Keywords:** postoperative pain, cigarette smoking, cerebrospinal fluid, beta-endorphin, substance P

## Abstract

**Objectives**: Cigarette smoking is associated with postoperative pain perception, which might be mediated by beta-endorphin and substance P. These effects on postoperative pain perception have never been investigated in human cerebrospinal fluid (CSF), which reflects biochemical alterations in the brain. Therefore, we investigated the associations among cigarette smoking, postoperative pain, and levels of beta-endorphin and substance P in human CSF.

**Methods**: We recruited 160 Chinese men (80 active smokers and 80 nonsmokers) who underwent lumbar puncture before anterior cruciate ligament reconstruction, and 5-ml CSF samples were collected. Pain visual analog scale (VAS) scores, post-anesthetic recovery duration (PARD), and smoking variables were obtained. CSF levels of beta-endorphin and substance P were measured.

**Results**: Compared to non-smokers, active smokers had significantly higher pain VAS (2.40 ± 0.67 vs. 1.70 ± 0.86, *p* < 0.001) and PARD scores (9.13 ± 2.11 vs. 7.27 ± 1.35, *p* = 0.001), lower CSF beta-endorphin (33.76 ± 1.77 vs. 35.66 ± 2.20, *p* = 0.001) and higher CSF substance P (2,124.46 ± 217.34 vs. 1,817.65 ± 302.14, *p* < 0.001) levels. Pain VAS scores correlated with PARD in active smokers (*r* = 0.443, *p* = 0.001).

**Conclusions**: Cigarette smoking is associated with increased postoperative pain intensity, shown by delayed pain perception, higher pain VAS scores, and lower beta-endorphin and higher substance P levels in the CSF of active smokers. The more extended postoperative pain perception is delayed, the more pain intensity increases.

## Introduction

Approximately 75% of patients who undergo surgery experience acute postoperative pain, which can persist for up to 7 days after surgery (Gupta et al., 2010). Severe persistent pain affects 2–10% of adults (Lespasio et al., 2019), and it can eventually become chronic, even with medication (Chapman and Vierck, 2017).

Evidence suggests that chronic exposure to cigarette smoke may exaggerate pain symptoms (Carstens et al., 2001; Anderson et al., 2004; Shi et al., 2010). A recent study showed that smokers take more oral morphine than nonsmokers (Howard et al., 2019), and smoking intensity is positively associated with chronic opioid use after surgery (Pang et al., 2017). Clinical studies reported that smokers require more pain medication for postoperative pain than nonsmokers or past smokers (Chiang et al., 2016). Nevertheless, several studies generated conflicting findings to the effect that cigarette smoke demonstrates an acute pain-inhibitory effect in humans and animal models (Shi et al., 2010; Ditre et al., 2011).

The relationship between pain and cigarette smoke is possibly mediated by changes in the endogenous opioid system (Shi et al., 2010). Beta-endorphin is an endogenous ligand for the mu-subtype opioid receptor (Sprouse-Blum et al., 2010) and is 18–33 times more potent as an analgesic than morphine (Loh et al., 1976). Animal studies showed that acute and chronic treatment with nicotine decreases beta-endorphin content in the hypothalamus, altering beta-endorphin synthesis and release in the brain (Gudehithlu et al., 2012). Patients undergoing general anesthesia experienced a significant increase in beta-endorphin during surgery, which was relieved after the co-administration of fentanyl (Dubois et al., 1982).

Chronic cigarette smoking enhances the expression of tachykinin receptors in the endothelium, mediating the effects of substance P (Zakharchuk et al., 2017). Substance P is a pro-nociceptive neuropeptide (a tachykinin) that mediates pain transmission (Stein, 1995; Snijdelaar et al., 2000; Hokfelt et al., 2003). Inhibiting substance P relieves pain (Zieglgansberger, 2019). Studies in transgenic mice showed that mice lacking substance P do not respond to moderate or severe pain (Hokfelt et al., 2003). Substantial evidence suggested that substance P plays an essential role in eliciting pain sensation in both the central nervous system (CNS) and the peripheral nervous system (PNS; Jasmin et al., 2002; Chang et al., 2019); cigarette smoking was found to promote neurogenic substance P release (Xu and Xu, 2010). In contrast to the role of beta-endorphin, substance P enhances pain sensation in the PNS, and when beta-endorphin binds to opioid receptors followed by a decrease in substance P levels, an analgesic state may result (Butler et al., 2015). These findings suggest that beta-endorphin and substance P may play different roles in postoperative pain intensity.

Neuropeptides like beta-endorphin and substance P cannot cross the blood-brain barrier (Riley et al., 2017), making the cerebrospinal fluid (CSF) an ideal medium to investigate biochemical alterations relating to pain in the human brain. This study used CSF to determine the association between pain intensity, beta-endorphin and substance P levels, and post-anesthetic recovery duration (PARD) in active smokers and explore the factors related to postoperative pain intensity.

## Materials and Methods

### Subjects

Because there are few female smokers in China, we, therefore, decided to exclude females from the study, and a total of 160 Chinese male subjects scheduled for anterior cruciate ligament single bundle reconstruction in the morning were recruited without surgery of meniscus repairing between September 2014 and January 2016. Of these, 80 were active smokers (the case group), and 80 were nonsmokers (the control group).

Sociodemographic data, including age, body mass index, and years of education, were collected. Clinical data, including substance abuse and dependence history, were obtained according to self-reporting and confirmed by the next of kin and family members. Exclusion criteria were as follows: (1) a family history of psychosis or neurological diseases, or CNS diseases determined using the Mini-International Neuropsychiatric Interview; and (2) systemic diseases based on the medical history and admitting diagnosis.

Subjects who had never smoked and had no history of substance abuse or dependence were assigned to the nonsmoker group. Active smokers were defined as those who had consumed half a pack of cigarettes (half pack = 10 cigarettes) or more per day for at least 1 year. Smokers that consumed fewer than 10 cigarettes per day were excluded from this study. Active smokers were further grouped into younger smokers (<40 years old) vs. older smokers (≥40 years old), according to the standard diagnostic manual of the American Psychiatric Association. Active smokers were divided into moderate smokers (*n* = 38, ≥10 and <20 cigarettes per day) and heavy smokers (*n* = 42, ≥20 cigarettes per day; the maximum was 40 cigarettes per day) according to the World Health Organization criteria. No subjects had a history of alcohol abuse or psychiatric disorders, according to the Diagnostic and Statistical Manual of Mental Disorders, fourth Edition. All subjects were unrelated.

The Institutional Review Board of the Inner Mongolian Medical University approved the study that was performed according to the Declaration of Helsinki. Written informed consent was obtained, and no financial compensation was provided to the subjects.

### Assessments, Biological Sample Collection, and Laboratory Tests

Information was obtained from active smokers, including the age of smoking onset, years of cigarette smoking, the average daily number of cigarettes smoked, and the maximum daily number of cigarettes smoked.

The data of high-density lipoprotein, low-density lipoprotein, alanine aminotransferase test, cholesterol, triglyceride, gamma-glutamyl transferase, and aspartate aminotransferase came from routine tests to evaluate physical condition on admission. These peripheral metabolic marker levels were measured in the morning on the 2nd hospital day after an overnight fasting period using a biochemistry analyzer (HITACH 7600, Hitachi Co., Tokyo, Japan).

Before surgery, the Self-rating Anxiety Scale (SAS) and the 13-item Beck Depression Inventory (BDI) were used to evaluate self-reported preoperative anxiety and symptoms of depression. An original raw score cutoff of 40 for SAS was identified previously (Dunstan and Scott, 2020). The ranges of BDI score cutoff points for various categories of depression were as follows: 0–4 (no depression), 5–7 (mild depression), 8–15 (moderate depression), and 16–39 (severe depression; Pomerleau, 1992). The interval between assessment and lumbar puncture was less than 24 h.

Lumbar puncture is part of normal clinical practice for patients undergoing anterior cruciate ligament reconstructive surgery in China, making the CSF sample conveniently accessible and decreasing the likelihood of disease entities affecting the CSF sample. Preoperative smoking cessation is not required for this kind of operation. In this study, 3 ml 0.5% ropivacaine was administered as local anesthesia in the morning before surgery by a licensed anesthetist, and a 5-ml CSF sample was obtained for analysis *via* intrathecal collection before ropivacaine was conducted.

It takes 40–50 min to complete anterior cruciate ligament reconstruction. Analgesics were not used before surgery, including opioid therapy and other medications; preoperative pain intensity was not recorded because there were no subjective painful symptoms. The anesthetist recorded the time of surgery completion, and the duty nurse recorded the time to postoperative pain perception. The duration between the two points was defined as PARD. Analgesics were not used after surgery. At the time of pain perception, the pain visual analog scale (VAS) was used to assess acute postoperative pain perception for all subjects. The scores ranged from 0 (“no pain”) to 10 (“most severe pain ever”).

Each CSF sample was then distributed into 0.5 ml tubes and immediately frozen at −80°C for storage. The period from hospitalization to surgery was a maximum of two days. CSF analyses to quantify beta-endorphin (Catalog#: RK-022-14) and substance P (Catalog#: RK-061-05) levels were performed using commercial radioimmunoassay kits from Phoenix (Phoenix Pharmaceuticals, Inc., Burlingame, CA, USA). The detection ranges of radioimmunoassay kits are 10–1,280 pg/ml. The cross reactivities of the peptides for beta-endorphin and substance P are both 100%. Deionized water of enzyme inactivation was set as the negative control for the assays. The positive control was included in the kits. All experimental procedures followed the manufacturer’s instructions, and the data analyst was blinded to the clinical data.

### Statistical Analysis

The normality of variance was performed using the Shapiro–Wilk test. The homogeneity of variance was performed using Levene’s test. Multi-collinearity was calculated using stepwise multiple regression among covariates estimated *via* tolerance and variance inflation factor (VIF) as the cutoff recommended thresholds for tolerance <0.1, and VIF >10 (Kim, 2019). Analysis of variance (ANOVA) and analysis of covariance (ANCOVA) were used to compare differences of variables between groups. All statistical analyses were performed using IBM SPSS Statistics for Windows, Version 22.0 (IBM Corp., Armonk, NY, USA). Figures were created using GraphPad Prism version 8 (GraphPad Software Inc., San Diego, CA, USA). All the tests were two-sided, and the significance threshold was set at *p* < 0.05.

## Results

### Demographic and Clinical Characteristics

Using Levene’s test, the homogeneity of variance was performed for sociodemographic and clinical variables (all *p* > 0.05). Consequently, ANOVA was used in Table 1.

The SAS raw scores and the BDI scores were ≤40 and ≤15, respectively, for all subjects. Nonsmokers had more years of education (13.33 ± 2.58 vs. 11.91 ± 3.16 years, *p* = 0.003), while older age and higher BDI scores were seen in active smokers (31.45 ± 9.03 vs. 34.71 ± 10.69 and 2.67 ± 3.12 vs. 0.79 ± 1.17, both *p* < 0.05). No differences were identified in age, SAS score, or clinical characteristics between the groups (all *p* > 0.05; Table 1).

**Table 1:** Differences in demographic and clinical characteristics between nonsmokers and active smokers.

Variables	Non-smokers (*n* = 80)	Active smokers (*n* = 80)	F (*df* =159)	*p*
Age (years)	31.45 ± 9.03	34.71 ± 10.69	4.35	0.039
Education (years)	13.33 ± 2.58	11.91 ± 3.16	9.41	0.003*
BMI (kg/m^2^)	25.26 ± 3.63	25.72 ± 3.52	0.66	0.418
SAS	33.46 ± 3.59	33.69 ± 4.28	0.12	0.725
BDI	0.79 ± 1.17	2.67 ± 3.12	24.27	<0.001*
High-density lipoprotein (mM/L)	1.25 ± 0.33	1.22 ± 0.28	0.38	0.537
Low-density lipoprotein (mM/L)	2.69 ± 0.55	2.67 ± 0.61	0.03	0.865
Alanine aminotransferase (U/L)	30.73 ± 20.80	31.79 ± 23.30	0.09	0.762
Cholesterol (mM/L)	4.75 ± 0.72	4.80 ± 0.82	0.16	0.689
Triglyceride (mM/L)	1.90 ± 0.90	1.69 ± 1.07	1.70	0.195
Gamma-glutamyl transferase (U/L)	44.35 ± 33.38	46.59 ± 44.42	0.13	0.881
Aspartate aminotransferase (U/L)	21.58 ± 8.35	20.66 ± 7.37	0.54	0.465

*Abbreviations: BMI, body mass index; SAS, Self—Rating Anxiety Scale; BDI, Beck Depression Inventory; ANOVA: analysis of variance; *df*, degree of freedom*. Note: All data are reported as mean ± standard deviation using ANOVA, *p < 0.05.

### Pain Perception and CSF Biomarkers

The normality of the residuals from the beta-endorphin and substance P models was determined using the Shapiro–Wilk test (*p* > 0.05). The homoscedasticity of residuals of the variances was determined using Levene’s test. The residuals were equally distributed (all *p* > 0.05). Stepwise multiple regression analyses of four pain-related variables, year, education, and BDI, showed that no variable was removed from models (all tolerance >0.8 and VIF <2). Because there was collinearity of age with age at smoking onset and years of cigarette smoking, respectively (tolerance < 0.1 and VIF > 30), age was removed from models in active smokers. Therefore, analysis of covariance ANCOVA was used to compare differences in pain-related variables between groups in Tables s 2, 3.

**Table 2:** Comparison of pain-related variables between nonsmokers and active smokers.

	Non-smokers (*n* = 80)	Active smokers (*n* = 80)	Mean differences	F (*df* =126)	95% CI	*p*
Pain VAS score	1.70 ± 0.86	2.40 ± 0.67	−0.85	19.33	−1.23, −0.47	<0.001*
PARD (hours)	7.32 ± 1.34	9.09 ± 2.15	−1.49	11.03	−2.38, −0.60	0.001*
CSF beta-endorphin (pg/ml)	35.47 ± 2.25	33.92 ± 1.80	1.81	12.13	0.78, 2.84	0.001*
CSF substance P (pg/ml)	1,849.98 ± 283.36	2,197.23 ± 209.55	−278.52	19.84	−402.35, −154.68	<0.001*

*Abbreviations: VAS, visual analog scale; PARD, post-anesthetic recovery duration; CSF, cerebral spinal fluid; BDI, Beck Depression Inventory; ANCOVA, analysis of covariance; *df*, degree of freedom*. Note: Data are reported as mean ± standard deviation using ANCOVA for each variable, with age, years of education, BDI, and the other variables in this table as covariates. *p < 0.05.

**Table 3:** The differences of CSF biomarkers levels between subgroups in active smokers.

	Younger/elder smokers (*n* = 57/23)				Moderate/heavy smokers (*n* = 48/32)			
	Mean differences	95% CI	F (*df* =56)	*p*	Mean differences	95% CI	F (*df* = 56)	*p*
Pain VAS score	0.09	−0.78, 0.97	0.47	0.42	0.09	−0.58, 0.76	0.06	0.81
PARD (hours)	−1.27	−4.03, 1.49	0.85	0.51	−0.76	−2.89, 1.37	0.50	0.48
CSF beta-endorphin (pg/ml)	1.48	−1.02, 3.97	1.43	0.80	−1.19	−3.06, 0.68	2.21	0.14
CSF substance P (pg/ml)	99.28	−45.97, 244.53	1.89	0.18	76.56	−161.26, 314.38	0.31	0.58

*Abbreviations: VAS, visual analog scale; PARD, post-anesthetic recovery duration; CSF, cerebral spinal fluid; BDI, Beck Depression Inventory; ANCOVA, analysis of covariance; *df*, degree of freedom*. *Note: ANCOVA was used for each variable, with years of education, BDI, smoking-related variables, and the other variables in this table as covariates*.

**Figure 1:**
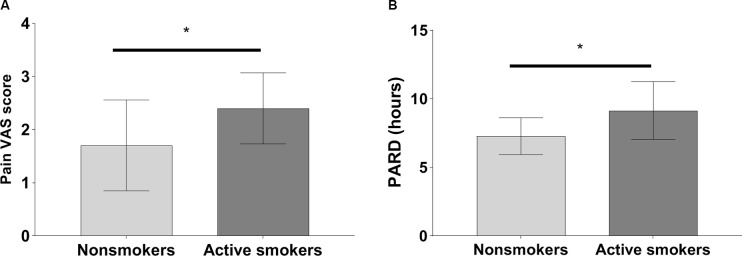
Significant differences in **(A)** pain visual analog scale (VAS) scores and **(B)** post-anesthetic recovery duration (PARD) between nonsmokers and active smokers (*p < 0.05).

**Figure 2:**
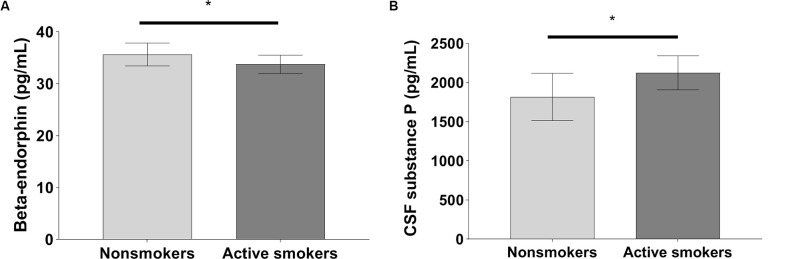
Significant differences in **(A)** cerebrospinal fluid (CSF) beta-endorphin levels, and **(B)** CSF substance P levels between nonsmokers and active smokers (*p < 0.05).

The pain VAS scores, PARD, and CSF levels of substance P in nonsmokers were remarkably lower than those in active smokers, while concentrations of beta-endorphin were significantly higher in nonsmokers when adjusting for years of education, BDI, and the other variables (all *p* < 0.05; Table 2, Figures 1, 2). ANCOVA was used for each variable, with years of education, BDI, smoking-related variables, and the other variables as covariates. There were no differences in pain VAS scores, PARD, or CSF levels of beta-endorphin and substance P between younger and elder smokers, moderate smokers, and heavy smokers, respectively (all *p* > 0.05; Table 3).

### Correlation of CSF Biomarkers and Smoking Status With Pain

**Figure 3:**
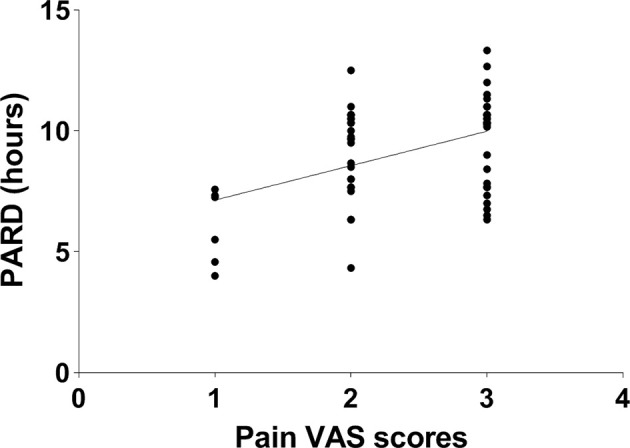
The pain visual analog scale (VAS) score was positively correlated with post-anesthetic recovery duration (PARD) in active smokers (*r* = 0.443, *p* = 0.001).

No correlation between CSF biomarkers and pain VAS scores or PARD was found in nonsmokers, adjusting for age, education, BDI, and other related variables (all *p* > 0.05). In active smokers, partial correlation analysis was performed between pain VAS scores and PARD (*r* = 0.443, *p* = 0.001, Figure 3), adjusting for years of education, BDI, and smoking-related variables.

Partial correlation analysis was performed to test the correlation between pain-related and smoking-related variables in active smokers, adjusting for education, BDI, other pain-related and smoking-related variables as covariates, and there was no correlation (all *p* > 0.05; Table 4).

**Table 4:** The correlation of CSF between pain-related and smoking-related variables in active smokers.

	Active smokers (*n* = 80)							
	Beta-endorphin (pg/ml		Substance P (pg/ml)		Pain VAS score		PARD (h)	
	r	p	r	p	r	p	r	p
Age of smoking onset	0.170	0.254	0.101	0.501	0.030	0.843	0.002	0.989
Years of cigarette smoking	0.182	0.222	0.087	0.561	0.028	0.850	−0.006	0.968
Number of cigarettes smoked /day	0.227	0.125	0.028	0.849	0.007	0.960	−0.055	0.712
Maximum number of cigarettes smoked/day	−0.041	0.785	−0.101	0.497	−0.099	0.508	0.143	0.339

*Abbreviations: VAS, visual analog scale; PARD, post-anesthetic recovery duration; CSF, cerebral spinal fluid; BDI, Beck Depression Inventory; ANCOVA, analysis of covariance*. *Note: Partial correlation analysis was performed for each variable, with education, BDI, other pain-related and smoking-related variables in this table as covariates*.

## Discussion

To the best of our knowledge, ours is the first study to use human CSF to calculate the association between cigarette smoking and postoperative pain. Our main finding is that cigarette smoking indeed increased postoperative pain intensity, with higher substance P and lower beta-endorphin levels in the CSF of active smokers than nonsmokers. Despite some conflicting results (Shi et al., 2010), several studies showed that cigarette smokers have more significant postoperative pain after total hip arthroplasty than a nonsmoking cohort (Etcheson et al., 2018). Furthermore, male active smokers require more morphine in the first 72 h after surgery than nonsmokers and past smokers (Chiang et al., 2016). Patients with smoking history were found to have increased analgesic intake compared to non-operative treatment (Forman et al., 2007). The administration of postoperative pain medication to coronary artery bypass graft patients showed that smokers require more pain medication than nonsmokers during the first 48 h after surgery (Creekmore et al., 2004). A prospective study demonstrated that smokers consume more opioids using patient-controlled analgesia during the first 24 h after surgery than nonsmokers (Steinmiller et al., 2012).

Beta-endorphin binds mu-opioid receptors at the presynaptic nerve terminals and inhibits the release of gamma-aminobutyric acid in the CNS, which inhibits the release of dopamine; the binding of beta-endorphin to these receptors leads to an overproduction of dopamine primarily associated with pleasure (Bressan and Crippa, 2005). Beta-endorphin relieves pain in adults (Stephan and Parsa, 2016) and fetuses (Lee et al., 2005). There are probably two functionally different beta-endorphin systems: one peripheral (release of beta-endorphin by the pituitary into the systemic circulation) and one central [synthesis in hypothalamic pro-opiomelanocortin (POMC) neurons]. Hypothalamic POMC neurons release beta-endorphin directly into the CSF of the third ventricle. The CSF serves as a transport medium for beta-endorphin to the distant brain and spinal sites. This is called “long-distance volume transmission” and is by no means unique for beta-endorphin. Hence, it makes physiological sense to speculate that low levels of CSF beta-endorphin would mirror a defective endogenous pain control system. Almay et al. reported that levels of unspecific “endorphins” were low in the CSF of patients with predominantly “neuralgic” pain, compared both to patients with what was labeled “psychogenic” pain and to healthy controls (Almay et al., 1978). Tonelli et al. (1988) found low CSF and beta-endorphin in patients scheduled for spinal cord stimulation, compared with historical controls. As a critical analgesic endogenous opioid (Bruehl et al., 2007), beta-endorphin is released from the pituitary gland (Veening et al., 2012) into the serum of humans (Bruehl et al., 2007) and mice (Rasmussen and Farr, 2003), following exposure to a painful stimulus. Previous studies have reported that patients undergoing general anesthesia experience a significant increase in beta-endorphin during surgery, inhibited by the co-administration of fentanyl (Cork et al., 1985). Similarly, patients who undergo dental surgery with local anesthesia have increased serum beta-endorphin levels during and after surgery. When fentanyl is co-administered, beta-endorphin levels in the serum are significantly reduced, and less pain is observed during the surgery (Hargreaves et al., 1983). All the studies mentioned above demonstrated the analgesic effect of beta-endorphin in the PNS.

Substance P is one of the main transmitters of nociceptive pain (Li et al., 2012). Substantial evidence suggests that substance P plays an essential role in eliciting pain sensation in both the CNS and PNS. In the CNS, substance P results in central sensitization by activating excitatory post-synaptic potential (De Koninck and Henry, 1991). In people with fibromyalgia, a chronic pain disorder, there are elevated substance P levels in CSF (Chang et al., 2019). Chemical ablation of neurons expressing substance P receptors in animals (Mantyh et al., 1997) and genetic disruption of the encoding gene of substance P or its receptor (Cao et al., 1998) reduced pain responses. A study demonstrated that 2 h after 2.5% formalin injection substance, P-expression in the distal CSF-contacting neurons was upregulated significantly, which may be considered essential for nociceptive processing (Lu et al., 2009). Together, these studies support the notion that substance P is an important signal molecule in pain transmission.

The evidence suggests higher substance P and lower beta-endorphin levels increase the intensity of postoperative pain, consistent with our results, as demonstrated by higher pain VAS scores after surgery. Studies reported that chronic exposure to nicotine and cigarette smoking reduces pain inhibition, agreeing with our findings (Carstens et al., 2001; Anderson et al., 2004; Simons et al., 2005). More importantly, ropivacaine is metabolized extensively by cytochrome P450 (CYP) enzymes (Arlander et al., 1998), and cigarette smoking increases the CYP-mediated metabolism of ropivacaine (Jokinen et al., 2001). CSF samples in our study were obtained before intrathecal injection of ropivacaine, which strongly indicates that smoking is more likely to enhance postoperative pain independently of ropivacaine.

Our secondary findings suggest delayed pain perception in active smokers and that pain VAS score positively correlated with PARD. First, cigarette smoking accelerates the metabolism of ropivacaine in the liver (Jokinen et al., 2001). Second, chronic cigarette smoking enhances tachykinin synthesis, stimulates neurogenic substance P release (Kwong et al., 2001; Xu and Xu, 2010), and accelerates the thinning of the brain’s cortex (Karama et al., 2015), which is one factor resulting in peripheral neuropathy (Richardson et al., 2009), such as axon loss and demyelination. Demyelinating neuropathy is characterized by a reduction in conduction velocity (Chung et al., 2014), which is also found in the sensory nerve (Walsh et al., 2015; Palve and Palve, 2018). Thus, this may be one reason for the longer duration in active smokers. Taken together, these findings suggest that cigarette smoking postpones postoperative pain. The evidence from these studies supports the notion that higher CSF substance P levels are found in active smokers with degenerating nerve conduction, further explaining why active smokers experience a higher pain intensity after a longer PARD. The positive correlation of pain VAS scores with PARD indicates that the longer the postoperative pain perception was delayed, the greater the pain intensity was. Since subjects were not required to stop smoking before or after surgery, withdrawal responses were not considered in the present study.

A study reported that the use of ropivacaine may reduce noxious input during surgery (Papaziogas et al., 2001); CSF samples were obtained prior to spinal anesthesia in the present study, which indicate the levels of beta-endorphin and substance P were the baseline levels and did not change due to surgery or ropivacaine. Thus, the CSF beta-endorphin and substance P levels can relate to postoperative pain in the present study. Moreover, active smokers who consumed less than half a pack of cigarettes (<10 cigarettes) per day, or had smoked for less than 1 year, were not included in our study, which might be another reason that no correlation was observed.

There are limitations to this study. First, we did not explore the immediate effect of cigarette smoking on pain perception because our subjects were all chronic cigarette smokers. Second, all subjects underwent surgery for anterior cruciate ligament injuries, which may not represent other types of postoperative pain. Thirdly, we did not record pack-year, since it was difficult to remember the amount for the subjects, though the common clinical quantification index of smoking exposure is pack-year. However, we collected smoking habit variables including the age of smoking onset, years of cigarette smoking, the average daily number of cigarettes smoked, and maximum daily number of cigarettes smoked instead. Finally, ropivacaine is metabolized extensively in the liver, predominantly by CYP, and its polymorphisms may play a role in the differences in ropivacaine metabolism. However, we did not detect the genetic polymorphisms of CYP in all subjects in the present study, which may be a slight confounder of our results, and genotyping these polymorphisms will be the subject of our future study.

## Conclusions

The relationship between cigarette smoking and postoperative pain is complex. Chronic cigarette smoking is associated with higher intensity postoperative pain, as shown by lower beta-endorphin and higher substance P levels in the CSF. The delay of postoperative pain perception correlates with greater pain intensity. These findings suggest that more analgesics should be considered for active smokers after surgery.

## Data Availability Statement

The raw data supporting the conclusions of this article will be made available by the authors, without undue reservation. Requests to access these datasets should be directed to Fan Wang, FanWang@bjmu.edu.cn.

## Ethics Statement

The studies involving human participants were reviewed and approved by Human Ethics Committee of Inner Mongolia Medical University. The patients/participants provided their written informed consent to participate in this study.

## Author Contributions

YL and K-PS provided the idea for the study. FW, K-PS, and YL contributed to the study design. FW and HL led the drafting of the manuscript and the statistical analyses. QM, YK, SY, LS, and H-CC collected the clinical data. H-CC helped edit the manuscript. All authors approved the final manuscript for submission.

## Conflict of Interest

The authors declare that the research was conducted in the absence of any commercial or financial relationships that could be construed as a potential conflict of interest.

## Publisher’s Note

All claims expressed in this article are solely those of the authors and do not necessarily represent those of their affiliated organizations, or those of the publisher, the editors and the reviewers. Any product that may be evaluated in this article, or claim that may be made by its manufacturer, is not guaranteed or endorsed by the publisher.
